# Development of new *Thermus thermophilus—Escherichia coli* shuttle vectors

**DOI:** 10.1128/aem.02102-25

**Published:** 2025-11-24

**Authors:** Sora Murayama, Haruki Omichi, Takumi Doi, Kentaro Miyazaki, Hiroya Tomita, Kohsuke Honda

**Affiliations:** 1 Graduate School of Engineering, The University of Osaka 13013, Suita, Osaka, Japan; 2 International Center for Biotechnology, The University of Osaka 13013, Suita, Osaka, Japan; 3 Industrial Biotechnology Initiative Division, Institute for Open and Transdisciplinary Research Initiatives (OTRI), The University of Osaka 13013, Suita, Osaka, Japan; Kyoto University, Kyoto, Japan

**Keywords:** compatibility, persistence, plasmid, replication protein, shuttle vector, synthetic biology, *Thermus thermophilus*

## Abstract

Genomic analysis revealed numerous plasmids in *Thermus thermophilus* strains isolated from the Senami Hot Spring in Japan. Five plasmids contained putative replication proteins (REP proteins) distinct from the pTT8-type commonly used in *T. thermophilus–Escherichia coli* shuttle vectors. Among them, two plasmids contained toxin-antitoxin-like tandemly aligned short open reading frames (ORFs) downstream of the putative REP protein. A series of shuttle vectors, pIOK, was constructed by cloning the putative REP protein gene and its flanking regions into an *E. coli* plasmid (ColE1, hygromycin-resistant). All five vectors were stably maintained in *T. thermophilus* HB27 in the presence of hygromycin. Among them, two containing the toxin-antitoxin-like module demonstrated greater persistence, exhibiting no plasmid loss after 168 generations of subcultivation without hygromycin. This module also enhanced the persistence of the pTT8-type shuttle vector pSN2, suggesting its broad applicability to the *Thermus* plasmids for stable maintenance. Quantitative PCR analysis demonstrated that the copy numbers of the pIOK vectors were equal to or greater than threefold those of the chromosomes. The pIOK vectors were compatible with one another and with pSN2. The xylan utilization pathway from *Thermus brockianus* (13 kbp) was split into two parts, which were cloned into pIOK and pSN2. Wild-type HB27 could not utilize xylan as the sole carbon source, while the double transformant carrying the two plasmids could, indicating the successful reconstitution of the complete xylan utilization pathway from two compatible plasmids. The pIOK vectors and toxin-antitoxin-like module will be invaluable for advancing synthetic biology research in *T. thermophilus*.

**IMPORTANCE:**

The rapid accumulation of genomic data from *Thermus thermophilus*, which we recently isolated from hot springs in Japan, has revealed the presence of plasmids with novel replication origins. We have developed a series of shuttle vectors, called pIOK, that are compatible with existing pTT8-based shuttle vectors. The effective use of multiple plasmid systems in *T. thermophilus* was demonstrated by dividing a relatively large (approximately 13 kbp) xylan assimilation pathway and reconstituting it using two compatible plasmids. The toxin-antitoxin-like module, located downstream of the newly identified replication proteins, significantly enhanced persistence, enabling the cultivation of the recombinant strain without the use of antibiotics. The pIOK vectors and the toxin-antitoxin-like module are expected to be valuable tools in the synthetic biology of *T. thermophilus*.

## INTRODUCTION

*Thermus thermophilus* (1, 2), which grows optimally at around 75°C, has been studied as a model thermophilic organism (3). Strain HB8 is used as a source for various biomacromolecules, such as proteins (4–8) and ribosomes (9–12), due to its extreme thermostability. Strain HB27 is mainly used in genetic studies (13–16) because of its high natural transformation efficiency and distinct colony formation on solid media. For genetic engineering purposes, various shuttle vectors have been developed that replicate in *T. thermophilus* and *Escherichia coli* (17–20). These vectors typically comprise ColE1-type replication origin for *E. coli*, a replication origin for *T. thermophilus*, and an antibiotic selection marker (20–24). Several replication origins are known for *T. thermophilus* plasmids (13, 18, 19); however, most shuttle vectors developed so far rely on the replication origin of pTT8, a 9.3 kbp plasmid found in HB8 (13, 20). In this era of synthetic biology, there is a demand for additional shuttle vectors for advanced genetic engineering in *T. thermophilus*, particularly those with replication origins that are compatible with the existing pTT8 type (18, 19).

Recently, dozens of new *T. thermophilus* strains were isolated from hot springs in Japan (25–32), and their complete genome sequences were determined. A survey of genomic data from these strains revealed that isolates from the Senami Hot Spring contained several small non-pTT8 type plasmids of approximately 20 kbp or less (31). In this study, a novel series of shuttle vectors, designated pIOK, was created by cloning a putative replication protein (REP protein) along with its flanking regions into the *E. coli* ColE1-type plasmid carrying a thermostable antibiotic resistance gene. The pIOK vectors were compatible with each other and with the pTT8-type plasmid. The effective use of multiple plasmid systems was demonstrated by dividing the relatively large (approximately 13 kbp) xylan utilization pathway into two parts, cloning each fragment into pIOK and pTT8-type vectors, and reconstituting xylan utilization ability *in vivo*.

## RESULTS AND DISCUSSION

### Construction of new *T. thermophilus***–***E. coli* shuttle vectors

We have recently isolated new *Thermus* strains from various hot springs in Japan: Arima (26, 30), Mine (25, 27, 28), Senami (31), and Shirahama (32). Complete genome sequencing revealed that five *T. thermophilus* strains isolated from Senami Hot Spring are rich in plasmids harboring at least one megaplasmid along with one to five other small plasmids, each approximately 20 kbp or smaller in size (31). No pTT8-type (13) plasmids were found.

**Fig 1:**
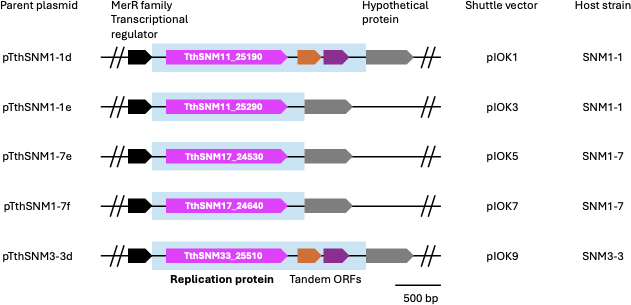
Schematic diagram of the putative replication protein and its flanking regions in five *T. thermophilus* plasmids isolated from Senami Hot Spring. The blue-shaded regions were PCR-amplified and cloned into pHSG298Hg.

**Fig 2:**
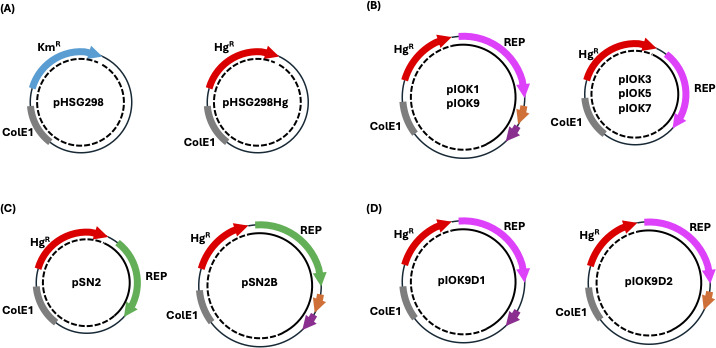
Schematic illustration of the vectors. (**A**) *E. coli* vectors. pHSG298 is a ColE1-based *E. coli* vector used as a backbone plasmid for all shuttle vectors constructed in this study. pHSG298Hg is a derivative of pHSG298 in which the kanamycin resistance gene (Km^R^) is replaced with a thermostable hygromycin resistance gene, *hph5*. (**B**) pIOK series *E. coli–T. thermophilus* shuttle vectors. pIOK1 and pIOK9 contain tandemly aligned short ORFs, the putative toxin-antitoxin module, downstream of the replication protein (also see Fig. 1). (**C**) pSN (pTT8-derivative) series *E. coli–T. thermophilus* shuttle vectors. (**D**) pIOK9 derivatives lacking one of the two ORFs in the putative toxin-antitoxin module.

We investigated the plasmid sequences and found highly conserved proteins (430-454 amino acids) in five plasmids: pTthSNM1-1d (locus tag, TthSNM11_25190), pTthSNM1-1e (TthSNM11_25290), pTthSNM1-7e (TthSNM17_24530), pTthSNM1-7f (TthSNM17_24640), and pTthSNM3-3d (TthSNM33_25510) (Fig. 1). The deduced amino acid sequences exhibited pairwise identities ranging from 83% to 95% (Table S1). Sequence variations, including point mutations and insertions/deletions, predominantly occurred in the N-terminal region (Fig. S1). A BLAST search using TthSNM11_25190 as a query yielded several homologous sequences in the NCBI (National Center for Biotechnology Information) database, but none were functionally characterized, and most were annotated as hypothetical proteins. We considered these genes to code for REP proteins and constructed a series of shuttle vectors by cloning the genes into the ColE1-type *E. coli* plasmid pHSG298Hg (Fig. 2A), in which the pre-encoded mesophilic kanamycin resistance gene in the commercial vector pHSG298 was replaced with the thermostable hygromycin resistance (Hg^R^) gene (or *hph5*) (22). In pTthSNM1-1d and pTthSNM3-3d, two short tandem open reading frames (ORFs) were identified ~100 bp downstream of the putative REP protein gene (TthSNM11_25200 and TthSNM11_25210 in pTthSNM1-1d, and TthSNM33_25520 and TthSNM33_25530 in pTthSNM3-3d) (Fig. 1) that were also included as targets for cloning. Note that the nucleotide sequences of the two ORFs and their intervening regions in pTthSNM1-1d and pTthSNM3-3d are identical. The target regions were PCR-amplified and inserted downstream of the *hph5* gene (Fig. 2B) to create plOK, a series of shuttle vectors (Fig. 2B). The detailed workflow for constructing the shuttle vectors is shown in Fig. S2.

In addition to the putative REP protein sequences, we also investigated the flanking regions. The 5′-untranslated regions (UTRs) of the REP protein (~50 bases) were classified into two groups: pTthSNM1-1e/pTthSNM1-7e/pTthSNM3-3d and pTthSNM1-1d/pTthSNM1-7f/pTT8 (Fig. S3A). It was unclear whether the conservation observed in each group was related to the replication mechanisms or simply attributed to an expression signal common to *T. thermophilus*. The 3′-UTR was fully conserved among pTthSNM1-1e, pTthSNM1-7e, and pTthSNM1-7f, while slight differences were observed in pTthSNM1-1d and pTthSNM3-3d (Fig. S3B). The difference between pTthSNM1-1d and pTthSNM3-3d and the others may be related to the occurrence of two ORFs downstream of the putative REP protein. No significant similarity was observed between pTthSNMs and pTT8.

### Replication test in *T. thermophilus*

We first tested the replicability of the shuttle vectors in *T. thermophilus*. The plOK3 vector was amplified in *E. coli* JM109 and introduced into *T. thermophilus* HB27 following the transformation procedure described previously (13). Colonies that appeared on TR/Hg agar plates (33) after overnight incubation at 70°C were inoculated in a liquid TR/Hg medium and cultivated at 70°C overnight. Plasmids were purified from the cells and reintroduced into *E. coli* JM109. After five rounds of host replacement, the plasmids were purified from *E. coli* cells and underwent whole plasmid sequencing. Despite the uneven distribution of G + C content (70.6% G + C for the *T. thermophilus* sequence compared to 53.5% for pHSG298Hg), no genetic rearrangements or mutations were observed, indicating stable vector replication in both *E. coli* and *T. thermophilus*.

### Plasmid persistence test and genetic characterization of the tandem ORF module

Next, we examined the persistence of the pIOK vectors in *T. thermophilus*. pSN2 (Fig. 2C), a pTT8-type shuttle vector, underwent the same analysis. First, the wild-type HB27 was transformed with each plasmid. Each transformant was grown in TR/Hg broth; HB27 was grown in TR without Hg. The growth curves were similar among clones (Fig. S4), and the doubling times were also similar: 1.02 h (pIOK1), 1.01 h (pIOK3), 1.03 h (pIOK5), 0.92 h (pIOK7), 1.05 h (pIOK9), 1.11 h (wild-type HB27), and 1.03 h (pSN2). As the doubling time for all clones was approximately 1 h, the number of generations was considered equal to the cultivation time. Transformed cells cultivated in the presence of Hg were then inoculated into fresh TR broth without Hg. Every 24 h, a portion of the sample was inoculated into fresh TR broth at a 1:100 dilution, while another portion was stored for later analysis. After 1 week of sub-cultivation, the stored cells were appropriately diluted and spread on TR or TR/Hg agar plates. After overnight cultivation at 70°C, the colonies on each plate were counted. Plasmid persistence was defined as the ratio of the number of colonies on TR plates to the number of colonies on TR/Hg plates.

**Fig 3:**
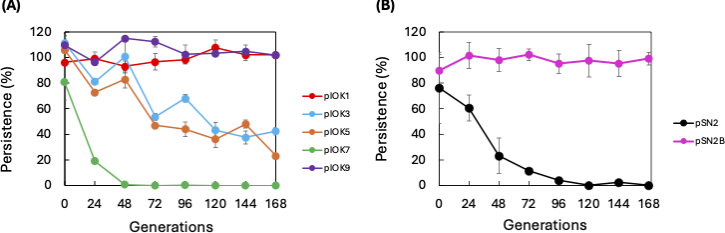
Plasmid persistence in *T. thermophilus*. (**A**) pIOK series. (**B**) pSN2 series. Transformed cells were cultivated at 70°C in TR broth (without Hg). The doubling time for each mutant was approximately 1 h (Fig. S2), and the number of generations was considered equal to the cultivation time. Every 24 h, a portion of the sample was inoculated into fresh TR broth at a 1:100 dilution, while another portion was stored for later analysis. After 1 week (or 168 generations) of sub-cultivation, the stocked cells were appropriately diluted and spread on TR or TR/Hg plates. After cultivation at 70°C overnight, the colonies on each plate were counted. Plasmid persistence was defined as the ratio of colony counts on TR plates to those on TR/Hg plates. Error bars represent standard errors calculated from triplicate measurements.

Among the five shuttle vectors, pIOK1 and pIOK9, which are equipped with tandem ORFs, displayed significantly greater persistence than the others, whereas pIOK7 exhibited the lowest persistence (Fig. 3A). pSN2 also showed low persistence (Fig. 3B). To investigate the role of the tandem ORF module in pIOK1 and pIOK9, the ORFs were cloned into pSN2, and the persistence of the resultant plasmid pSN2B (Fig. 2C) was assessed in the same manner as with the pIOK vectors. As shown in Fig. 3B, the persistence improved significantly, comparable to that of pIOK1 and pIOK9, indicating the general role of the tandem ORFs in plasmid persistence. The tandem ORFs, henceforth referred to as the “Persistence-Enhancing Module (PEM),” underwent further genetic analysis to explore the role of each ORF.

Given its enhancing effect on plasmid persistence and the unique genetic architecture, we hypothesized that the tandem ORFs form a type II toxin-antitoxin system (34, 35). The two short ORFs were categorized as DUF4160 domain-containing protein (87 amino acids) and DUF2442 domain-containing protein (92 amino acids) in the UniProt database; however, their detailed biological functions remain unclear. To investigate the role of these ORFs, one of the two ORF genes was deleted from pIOK9 to generate pIOK9D1 (for the first ORF deletion) and pIOK9D2 (for the second ORF deletion) (Fig. 2D). Both plasmids were stably maintained in *E. coli* (data not shown), implying that these ORFs have no physiological impact on *E. coli*. However, when attempts were made to introduce each plasmid into *T. thermophilus* HB27, Hg^R^ transformants were obtained with pIOK9D1 but not with pIOK9D2. The results suggest that the second ORF neutralized the toxic effect of the first ORF.

**Fig 4:**
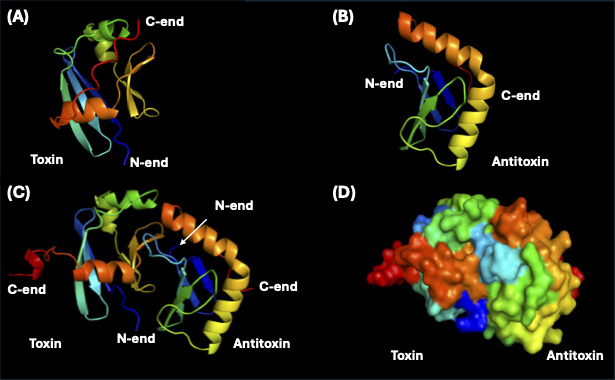
Predicted three-dimensional structures of putative toxin and antitoxin proteins. (**A**) Toxin monomer. (**B**) Antitoxin monomer. (**C**) Toxin-antitoxin heterodimer (cartoon model). (**D**) Toxin-antitoxin heterodimer (surface model). The three-dimensional structures were predicted by AlphaFold 3 (36).

The three-dimensional structure of the putative toxin-antitoxin module was predicted by AlphaFold 3 (36). The predicted three-dimensional structures of each monomer are shown in Fig. 4A (putative toxin) and Fig. 4B (putative antitoxin). When the two protein sequences were input together for prediction, they formed a heterodimer (cartoon model in Fig. 4C; surface model in Fig. 4D), suggesting an intimate interaction between the two proteins. The predicted structure of monomeric antitoxin was slightly different from that of the complex at the N-terminus region, while the structure of the toxin changed significantly at the C-terminus. Although the detailed biochemical functions remain unclear, all the above-mentioned characteristics strongly suggest that the two ORFs form a type II toxin-antitoxin system. The high persistence observed in the shuttle vectors pIOK1, pIOK9, and pSN2B is likely associated with this type II toxin-antitoxin system.

### Copy number estimation

The copy numbers for chromosomes and pIOK vectors were estimated through qPCR analysis. The *polI* gene, which encodes DNA polymerase I, a chromosomally coded single-copy gene, was targeted to measure the chromosome copy number, whereas the Hg^R^ gene was targeted to measure the plasmid copy number. Note that *T. thermophilus* is a polyploid bacterium containing 4-5 copies of chromosomes per cell (37). Therefore, in this study, the plasmid copy number was expressed relative to the chromosome copy number. As shown in Table 1, the relative copy numbers of the pIOK vectors ranged from 1.22 to 3.29, which were higher than that of the pTT8-type shuttle vector pSN2 (0.79). Given that *T. thermophilus* has 4-5 chromosome copies per cell (37), our calculations indicate that each cell contains a total of 5–16 copies of pIOK vectors. The copy number of the pTT8-derived shuttle vector pSN2 seems lower than that of the original pTT8 (8 [38] versus 4-5 [37]). However, the conditions for the copy number measurement (cultivation, DNA extraction, analytical methods) are significantly different, and the genomic backgrounds also differ, making direct comparison difficult. Further studies will be needed to resolve the apparent discrepancy.

**TABLE 1:** Copy numbers of shuttle vectors

Shuttle vector	Copy number^*a*^
pIOK1	1.22 ± 0.04
pIOK3	3.29 ± 0.27
pIOK5	3.29 ± 0.16
pIOK7	2.16 ± 0.09
pIOK9	2.69 ± 0.22
pSN2	0.79 ± 0.04

^*a*^ Values represent the copy numbers relative to that of chromosomes. Note that *T. thermophilus* is a polyploid and contains four to five copies of chromosomes per cell (37). Errors represent standard errors calculated from triplicate measurements.

### Compatibility test

The five REP proteins encoded by pIOK vectors have generally been treated as a single group, owing to their high sequence similarity (>83%) (Table S1). However, in the original isolates, multiple plasmids coexist within the same strain: pTthSNM1-1d and pTthSNM1-1e (together with three additional plasmids) in strain SNM1-1, and pTthSNM1-7e and pTthSNM1-7f (together with four additional plasmids) in strain SNM1-7. These naturally coexisting plasmids are, in theory, expected to be mutually compatible.

To experimentally evaluate compatibility among the five pIOK shuttle vectors (with similar but not identical REP protein sequences), the Hg^R^ marker (*hph5*) (22) in each pIOK vector was replaced with a thermostable Km^R^ (*htk*) (21), yielding a corresponding series of derivatives designated pMAO. Next, to maintain one of the two plasmids (and to see the persistence of the second plasmid), the Km^R^/Hg^R^ double transformants were propagated in TT medium containing Km alone. Under these conditions, the Km^R^ plasmid is forced to be maintained, while the coexisting Hg^R^ plasmid is released from selection pressure. If the two plasmids are incompatible, the Hg^R^ plasmid will be rapidly lost. In contrast, if they are compatible, the Hg^R^ plasmid will be maintained or lost depending on its native persistence.

First, we tested the compatibility between pMAO1 (Km^R^; pIOK type) and pSN2B, a derivative of pSN2 (Hg^R^; pTT8 type) carrying the PEM. The results showed that in the presence of Km alone, pSN2B was retained at 93% after 24 generations and 80% after 96 generations (Fig. S5). Although a slight loss of pSN2B was observed, we considered that this is due to the growth competition between the strain harboring two plasmids and the strain harboring a single Km^R^ pMAO1 alone (relative fitness ~0.997/generation, accounting for the 93% retention after 24 generations: 0.997^24^ = 0.93), but not due to the plasmid incompatibility, which should have a much heavier impact on the persistence.

Similar experiments were conducted with pIOK9 (Hg^R^)–pMAO1 (Km^R^) pair. pIOK9 was retained at 99% after 24 generations and 95% retention after 96 generations (Fig. S5). As with the pSN2B and pMAO1 pair, very minor fitness loss (relative fitness ~0.9995/generation) was observed, which may also be well explained by the same reason for the pMAO1 and pSN2B as described above.

Collectively, these results indicate that the pTT8 and pIOK types are compatible, and that plasmids within the pIOK series are also mutually compatible, consistent with their stable coexistence in natural microbial communities.

### Functional reconstitution of the xylan-utilization pathway using two compatible plasmids

**Fig 5:**
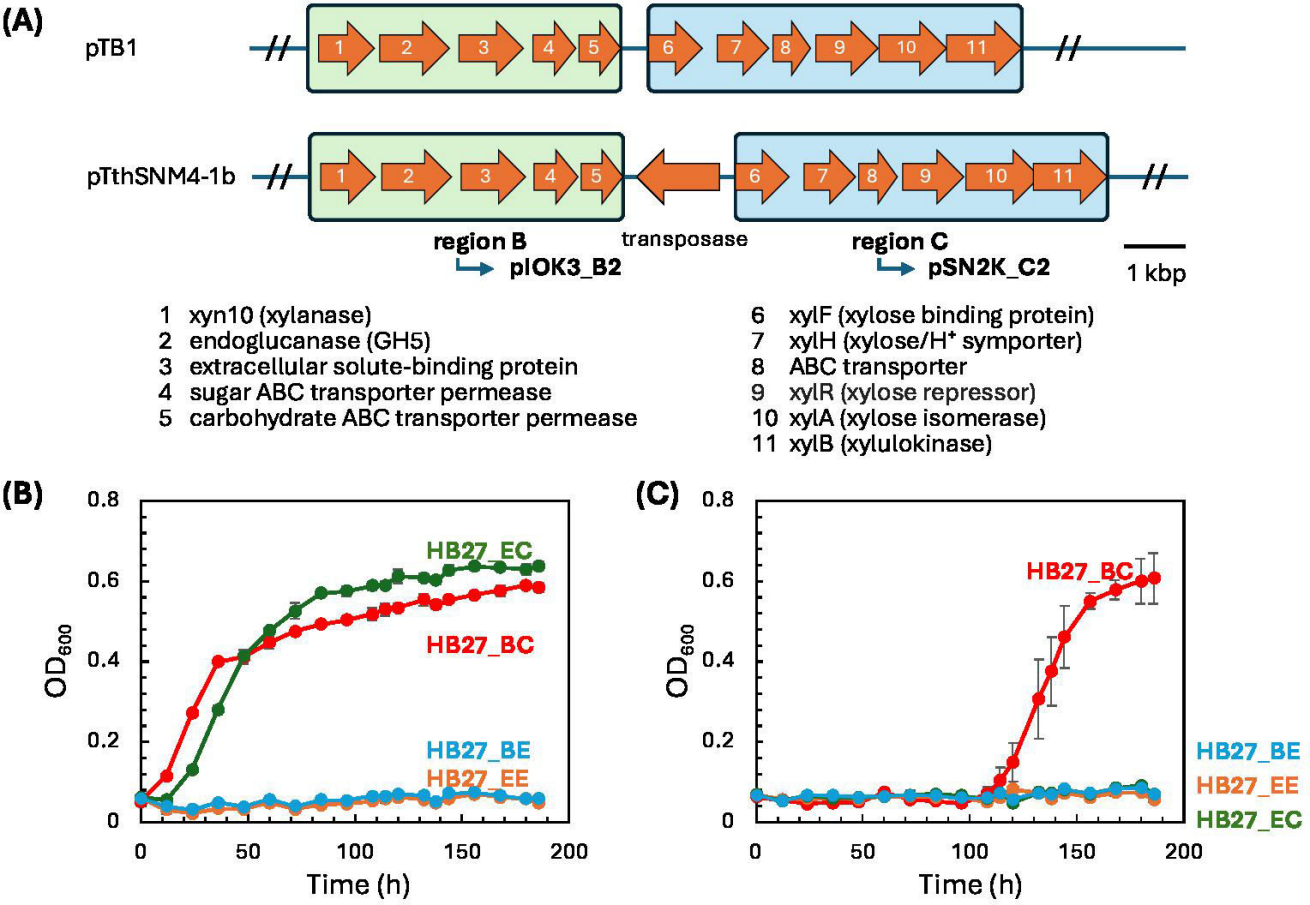
Xylan utilization pathway in *T. brockianus* SNM4-1. (**A**) The xylan utilization pathways identified in *T. brockianus* strains GE-1 (pTB1) and SNM4-1 (pTbrSNM4-1b). In the SNM4-1 strain, the pathway is interrupted by the transposase gene. The upper pathway genes (region B) are thought to be involved in xylan degradation (39). The lower pathway genes (region C) are thought to be involved in xylose assimilation (39). The bipartite fragments were cloned into pIOK3 (Hg^R^) and pSN2K (Km^R^) to generate pIOK3_B2 and pSN2K_C2, respectively. (**B**) Growth curves of the double-transformants in the minimum medium supplemented with xylose as the sole carbon source (and Hg and Km). (**C**) Growth curves of the double-transformants in the minimum medium supplemented with xylan as the sole carbon source (and Hg and Km). Strain annotations for (**B**) and (**C**): HB27_EE, HB27 carrying empty vectors, pIOK3_B2_Emp and pSN2K_C2_Emp; HB27_EC, HB27 carrying pIOK3_B2_Emp and pSN2K_C2; HB27_BE, HB27 carrying vectors, pIOK3_B2 and pSN2K_C2_Emp; HB27_BC, HB27 carrying vectors, pIOK3_B2 and pSN2K_C2.

Taking advantage of the compatibility between pIOK and pSN, we next cloned a relatively large genomic fragment into two plasmids by splitting the long fragment into two parts. We targeted the xylan assimilation pathway genes coded in the pTbrSNM4-1b, a megaplasmid (~350 kbp) housed in *Thermus brockianus* SNM4-1 (Fig. 5A). Unlike *T. brockianus* GE-1 (39), which carries a consecutive ~13 kbp xylan-utilization pathway, SNM4-1 has an intervening transposase gene (TbrSNM41_23860) that splits the xylan-utilization pathway into two segments. Because the entire pathway spans 15 kbp, amplifying this long target was a challenging task. Therefore, we divided the pathway into two parts: the transposase gene was excluded from the cloning process. The upper region was 6.0 kbp (region B), while the lower region was 7.3 kbp (region C). The upper region contains the xylanase enzyme, and the lower region is thought to contain enzymes involved in xylose metabolism. The two fragments were PCR-amplified and cloned into pIOK3 and pSN2K to generate pIOK3_B2 and pSN2K_C2, respectively (Fig. 5A; Fig. S6 and S7). HB27 was then transformed using the following combinations: (i) pIOK3_B2_Emp (empty vector) and pSN2K_C2_Emp (empty vector), the recombinant strain named HB27_EE; (ii) pIOK3_B2_Emp and pSN2K_C2 (HB27_EC); (iii) pIOK3_B2 and pSN2K_C2_Emp (HB27_BE); and (iv) pIOK3_B2 and pSN2K_C2 (HB27_BC). When these transformants were cultivated in a minimal medium containing xylose (with Hg and Km supplementation) (Fig. 5B) as the sole carbon source, growth was observed in HB27_EC and HB27_BC (Fig. 5B). The results indicate that region C is responsible for xylose utilization. When these transformants were cultivated in a minimal medium containing xylan as the sole carbon source (with Hg and Km supplementation), growth was observed only in HB27_BC (Fig. 5C). These results imply the successful functional reconstitution of the complete pathway using the two compatible plasmids.

### Concluding remarks

We constructed a series of shuttle vectors, pIOK, using plasmids discovered in *T. thermophilus* strains isolated from Senami Hot Springs in Japan. We verified plasmid compatibility with pTT8-type vectors and also confirmed mutual compatibility among the pIOK vectors. Two vectors, pIOK1 and pIOK9, carrying the putative toxin-antitoxin module, which was named the Persistence Enhancing Module (PEM), showed higher persistence than the other pIOK vectors. pSN2, a pTT8-type shuttle vector, was quickly lost in the absence of antibiotics but remained long in the cell in the presence of PEM. The pIOK vectors and PEM have the potential to be valuable genetic tools to advance synthetic biology research in *T. thermophilus*.

## MATERIALS AND METHODS

### Reagents

Hipolypeptone N, agar, hygromycin B (Hg), and kanamycin (Km) were purchased from Wako Pure Chemical/FUJIFILM (Osaka, Japan). D-Xylose and Lennox LB (1% [wt/vol] tryptone, 0.5% [wt/vol] yeast extract, 0.5% [wt/vol] NaCl) were purchased from Nacalai Tesque (Kyoto, Japan). DpnI, T4 DNA polymerase, T4 DNA ligase, and NEBuilder HiFi DNA Assembly Master Mix were purchased from New England Biolabs (Ipswich, MA, USA). pHSG298 was purchased from TakaraBio (Shiga, Japan). Oligonucleotides were purchased from Eurofins Genomics (Tokyo, Japan). Oat spelt xylan was purchased from Megazyme (Bray, Ireland).

### Bacterial strains and culture conditions

*T. thermophilus* strains (laboratory stocks) were routinely cultivated in TR medium (8 g L^-1^ hiploypeptone N, 4 g L^-1^ yeast extract, and 2 g L^-1^ NaCl), TT medium (TR medium containing 0.4 mM each of MgCl_2_ and CaCl_2_) (21), or the minimal medium for *T. thermophilus*, which consisted of 0.5 g L^-1^ K_2_HPO_4_, 0.25 g L^-1^ KH_2_PO_4_, 0.25 g L^-1^ NaCl, 0.5 g L^-1^ (NH_4_)_2_SO_4_, 0.125 mg L^-1^ MgCl_2_·6H_2_O, 0.025 g L^-1^ CaCl_2_·2H_2_O, and 1/100 vol of Thermophilus Vitamin-Mineral Stock 100X (United States Biological, Salem, MA, USA). Competent *E. coli* JM109 cells were purchased from RBC Bioscience (Taipei, Taiwan). *E. coli* strains were cultivated in LB medium at 37°C. Antibiotics (Hg and Km) were added at 100 µg mL^-1^ when necessary. For solid media, agar (16 g L^-1^) or gellan gum (8 g L^-1^) was used. To solidify gellan gum plates, 4 mM each of MgCl_2_ and CaCl_2_ was added to the medium. Growth curves were generated from the OD_660_ values measured using a Taitec (Saitama, Japan) Miniphoto 518R.

### General techniques

PCR was carried out using KOD FX Neo DNA polymerase (Toyobo, Osaka, Japan) with the following temperature cycle: initial denaturation at 98°C for 2 min, followed by 30 cycles of incubation at 98°C for 10 s, 57°C for 30 s, and 68°C for xx s (xx: extension time adjusted according to the target length, 30 s/kbp), followed by a final incubation at 68°C for 1 min. Gibson assembly (40) was performed using NEBuilder HiFi DNA Assembly Master Mix following the manufacturer’s instructions. PCR products were purified using the FastGene Gel/PCR Extraction Kit (Nippon Genetics, Tokyo, Japan). Plasmids were purified using the FastGene Plasmid Mini Kit (Nippon Genetics). All vectors constructed in this study were validated by DNA sequencing.

### Construction of new shuttle vectors

The genomic and plasmid DNA mixture was purified as described (31). The gene for the putative REP protein, along with its flanking regions (Fig. 1), was PCR-amplified for each strain (TthSNM1-1, TthSNM1-7, and TthSNM3-3) using the primers listed in Table S2. pTthSNM1-1e, pTthSNM1-7e, and pTthSNM3-3d shared identical sequences at the 5′ end of the target sequence, so a common forward primer (IOK359Fw) was used for these plasmids. For pTthSNM1-1d and pTthSNM1-7f, IOK1Fw and IOK7Fw were used as forward primers, respectively. A common reverse primer (IOKRv) was used for all targets. For the vector, a commercial ColE1-based pHSG298 plasmid was first modified by replacing the mesophilic Km^R^ gene with a thermostable Hg^R^ gene, *hph5* (22), to yield pHSG298Hg. The entire pHSG298Hg plasmid was then amplified by inverse PCR using a set of primers, HgRv and Kmdel2. The product was treated with DpnI (10 U) at 37°C for 2 h to eliminate the template. The vector and insert fragments were agarose gel-purified and then ligated by Gibson Assembly (40). Competent *E. coli* JM109 cells were transformed with the reaction mixture, and transformants were selected on LB/Hg plates at 37°C. The resultant shuttle vectors, carrying the genes for the putative replication proteins and their flanking regions of *T. thermophilus* plasmids downstream of the *hph5* gene, were named pIOK# (# denotes serial numbers) (Fig. 2B).

pMAO vectors are the derivatives of pIOK in which the *hph5* (22) gene was replaced by a thermostable Km^R^ gene, *htk* (21). The Km^R^ gene was amplified using the KmR_Fw and KmR_Rv primers; the pIOK vector was amplified using Kmdel1_KmRFw_gib (common to all) and I1ori_Fw_Kmgib (pMAO1), I359ori_Fw_Kmgib (pMAO3, 5, 9), or I7ori_Fw_Kmgib (pMAO7). The fragments were ligated by Gibson Assembly (40), and the proper construction of the desired plasmids was confirmed by DNA sequencing.

Similarly, the replication origin of pTT8 (GenBank ID: NZ_AP024986.1) was PCR-amplified using a set of primers, SN2Fw and SN2Rv, from pTT8 and cloned into the same site of pHSG298Hg to produce pSN2. pSN2 (Fig. 2B), a pTT8-type shuttle vector, was used as a control. Note that the orientation of pTT8-Rep differs from that of pIOK, as we could not obtain the vector in the same direction as pIOK, likely due to plasmid instability in *E. coli*. pSN2K was constructed by replacing the *hph5* gene with the *htk* gene (21) as follows. The *htk* gene was amplified using primers KmR_Fw and KmR_Rv; the vector was amplified using Kmdel1_KmRFw_gib and N2ori_Fw_Kmgib. pSN2B was constructed by cloning the tandem ORFs (TthSNM33_25520 – TthSNM33_25530) downstream of the pTT8 Rep. To this end, pSN2 was inversely amplified using reppTT8_1397F and repA_Rv primers, while the tandem ORFs were amplified from pIOK9 using hps_Fw_reppTT8_gib and hps_Rv_reppTT8_gib primers (primers listed in Table S3). Each amplicon was gel-purified and ligated by Gibson Assembly (40).

Two derivative plasmids, pIOK9D1 and pIOK9D2, lacking the first and second ORFs (locus tags, TthSNM33_25520 and TthSNM33_25530, respectively) located downstream of the putative Rep protein (TthSNM33_25510) in pIOK9 (Fig. 2B), were constructed by inverse PCR. Sets of primers repSNM3-3d_1809F and repSNM3-3d_1457R were used for pIOK9D1, and repSNM3-3d_2095F and repSNM3-3d_1818R for pIOK9D2 (primers listed in Table S4). The amplicons were agarose-gel purified and self-ligated using T4 DNA polymerase and T4 DNA ligase. Correct construction of the desired plasmids was confirmed by DNA sequencing. Sanger sequencing and Nanopore-based whole plasmid sequencing were outsourced to AZENTA (Tokyo, Japan).

### Copy number measurement

The copy numbers of the chromosome and shuttle vectors were assessed using qPCR. *T. thermophilus* strains transformed by pIOK vectors were cultivated at 70°C in TR/Hg broth until OD_660_ reached 1.1–1.3. The mixture of chromosome and plasmid DNA was purified using the KANEKA Easy DNA Extraction Kit version 2 (Kaneka Corporation, Osaka, Japan). qPCR was conducted using the KAPA SYBR Fast qPCR Kit (Kapa Biosystems, Wilmington, MA, USA). The *hph5* gene was targeted to measure the plasmid copy number, while the *polI* gene, encoding DNA polymerase I (41), was targeted to measure the chromosome copy number. The primers were designed using the Primer3 software (42) with the following parameter settings: an optimal annealing temperature, 60°C; G + C contents, 50%; amplicon size, 100–200 bp (primers listed in Table S5). The qPCR was performed using the following temperature cycle: 95°C for 10 min, followed by 40 cycles of incubation at 95°C for 15 s and 60°C for 40 s, on a QuantStudio 3 real-time PCR system (Thermo Fisher Scientific, Waltham, MA, USA). The melt curve analysis ensured that a single product was amplified. The C_T_ values for standards and samples were measured in triplicate, and the amount of DNA was calculated using the standard curves.

### Compatibility tests

Shuttle vectors pMAO1 (Km^R^) and pSN2B (Hg^R^) or pMAO1 (Km^R^) and pIOK9 (Hg^R^) were co-introduced into *T. thermophilus* HB27, and double transformants were selected on TT/Km/Hg plates. For each combination, a single colony was used for compatibility tests. A single colony was inoculated in 5 mL of TT/Km medium at 65°C. Every 24 h, a portion of the culture was inoculated into a fresh 5 mL of TT/Km medium at a 1:1,000 dilution, while another portion was appropriately diluted (~10⁵) and spread on TT/Km plates. After overnight cultivation at 65°C, 100 colonies were randomly selected and replica-plated onto TT/Km and TT/Km/Hg plates. The number of colonies on TT/Km was taken as 100%, and the number of colonies on TT/Km/Hg was used to calculate the rate of compatibility.

### Construction of expression vectors for the xylan utilization pathway

The upper pathway (region B) was PCR-amplified using primers xylanase_up2_F2 and half_2000_1R (2,174 bp), colopyxylan4F and half_2000-2R (2,138 bp), and half_2000_3F and IOK3_B_kaiF (1,737 bp) from the genomic DNA of *T. brockianus* strain SNM4-1 (primers listed in Table S6). The three fragments carrying overlapping ends were ligated with a linearized pIOK3, prepared by inverse PCR using primers pSN1_Fw and IOK3_B_kaiR, using Gibson Assembly (40). The resultant plasmid was named pIOK3_B2. To create a negative control plasmid (empty vector), inverse PCR was performed using primers pSN_1_Fw and IOK_nega_R. The fragment was self-ligated using Gibson Assembly (40). The resultant plasmid was named pIOK3_B2_Emp. A schematic workflow of the construction of pIO3_B2 and pIOK3_B2_Emp is shown in Fig. S6.

The lower pathway (region C) was PCR amplified using primers xylanase_pSN_F and xylB_pSN_R from the genomic DNA of *T. brockianus* strain SNM4-1. The fragment was ligated with a linearized pSN2K, prepared by inverse PCR using primers pHSG298Hg_1F and pHSG298Hg_1R, using Gibson Assembly (40). The resultant plasmid, pSN2K_C1, was further modified to replace the 5′ UTR region of cds-1 with the strong promoter, nqo3 (43). The nqo3 promoter was amplified using primers pSN_C_nqo_F and pSN_C_nqo_R using pMotK3A as a template (43). The fragment was ligated with a linearized pSN2K_C1, prepared by inverse PCR using primers pSN_C_inverse_F and pSN_C_inverse_R, using Gibson Assembly (40). The resultant plasmid was named pSN2K_C2. To construct a negative control plasmid (empty vector), inverse PCR was performed using primers nqo_nega_F and nqo_reverse. The fragment was self-cloned by Gibson Assembly (40). The resultant plasmid was named pSN2K_C2_Emp. A schematic workflow for constructing pSN2K_C1, pSN2K_C2, and pSN2K_C2_Emp is shown in Fig. S7.

### DNA analyses

Multiple sequence alignment was performed using the MUSCLE v5.3 program (44) implemented in Geneious Prime (GraphPad Software, Boston, MA).

## Data Availability

DNA sequence data for the vectors developed in this study have been deposited in the NCBI database under the accession numbers (LC895482–LC895503) listed in Table S7.

## SUPPLEMENTAL MATERIAL

Supplemental material aem.02102-25-s0001.pdf Tables S1 to S7; Fig. S1 to S7.

ASM does not own the copyrights to Supplemental Material that may be linked to, or accessed through, an article. The authors have granted ASM a non-exclusive, world-wide license to publish the Supplemental Material files. Please contact the corresponding author directly for reuse.
