# Supplementary material for: Development of new Thermus thermophilus—Escherichia coli shuttle vectors
Source: Appl Environ Microbiol. 2025 Nov 24;91(12):e02102-25. doi: 10.1128/aem.02102-25 (PMC12724237; doi:10.1128/aem.02102-25)
Supplement: Supplemental material — Tables S1 to S7; Fig. S1 to S7. [file aem.02102-25-s0001.pdf]

Table S1 Pairwise amino acid sequence identities between the replication proteins

	TthSNM11_25190	TthSNM17_24640	TthSNM11_25290	TthSNM17_24530	TthSNM33_25510
TthSNM11_25190	100	94.5	82.2	85	86.3
TthSNM17_24640	94.5	100	81.5	87	83.3
TthSNM11_25290	82.2	81.5	100	93.6	95.4
TthSNM17_24530	85	87	93.6	100	95.4
TthSNM33_25510	86.3	83.3	95.4	95.4	100

Table S2 Oligonucleotide primers used for the construction of shuttle vectors

Primer	Sequence (5' to 3')	Note
IOK1Fw	TCCGGAGCCGGGTGACCTGGGGACTACCGC	Forward primer for the replication origin in plasmid pTthSNM1-1d
IOK359Fw	TCCGGAGCCGGGTGAGGGGCCTTAGGAAAG	Forward primer for the replication origins in plasmids pTthSNM1-1e, pTthSNM1-7e, and pTthSNM3-3d
IOK7Fw	TCCGGAGCCGGGTGAGGGGGGATTGGGTGG	Forward primer for the replication origin in plasmid pTthSNM1-7f
IOKRv	ATTAACCAATTCTGAGGGGTCCAGGGTAAG	Reverse primer for replication origins in plasmids pTthSNM1-1d, pTthSNM1-1e, pTthSNM1-7e, pTthSNM1-7f, and pTthSNM3-3d
HgRv	TCACCCGGCTCCGGATCGGACGATT	Inverse PCR primer to delete antibiotic resistance gene from pHSG298
Kmdel2	TCAGAATTGGTTAATTGGTTGTAAC	Inverse PCR primer to delete antibiotic resistance gene from pHSG298
KmR_Fw	ATGAAAGGACCAATAATAATGACTAGAGAA	Forward primer for the thermostable Km^R gene (*htk*)
KmR_Rv	TCATCAAAATGGTATGCGTTTTGACACATC	Reverse primer for the thermostable Km^R gene (*htk*)
Kmdel1_KmRFw_gib	TATTGGTCCTTTCATAACACCCCTTGTATTACTGTTTATG	Inverse PCR primer (upstream of the Km^R gene) for inserting the thermostable Km^R gene (*htk*) into pMAO (common)
I1ori_Fw_Kmgib	ATACCATTTTGATGACCTGGGGACTACCGC	Inverse PCR primer (downstream of the Km^R gene) for inserting the thermostable Km^R gene (*htk*) into pMAO1
I359ori_Fw_Kmgib	ATACCATTTTGATGAGGGGCCTTAGGAAAG	Inverse PCR primer (downstream of the Km^R gene) for

		inserting the thermostable Km^R gene (*htk*) in pMAO3, 5 ,9
I7ori_Fw_Kmgib	ATACCATTTTGATGAGGGGGGATTGGGTGG	Inverse PCR primer (downstream of the Km^R gene) for inserting the thermostable Km^R gene (*htk*) in pMAO7
SN2Fw	TCCGGAGCCGGGTGAAGGGCGGGGCACCCG	Forward primer for the replication origin in plasmid pTT8
SN2Rv	ATTAACCAATTCTGACCTGGGGACTACCGCTTGGT	Reverse primer for the replication origin in plasmid pTT8
Kmdel1_KmRFw_gib	TATTGGTCCTTTCATAACACCCCTTGTATTACTGTTTATG	Inverse PCR primer (upstream of the Km^R gene) for inserting the thermostable Km^R gene (*htk*) in pSN2
N2ori_Fw_Kmgib	ATACCATTTTGATGAAGGGCGGGGCACCCG	Inverse PCR primer (downstream of the Km^R gene) for inserting thermostable Km^R gene (*htk*) in pSN2

Table S3 Oligonucleotide primers used for the construction of pSN2B

Primer	Sequence (5’ to 3’)	Note
reppTT8_1397F F	CGTCCAGGACGGCACAAACCCCAAA	Inverse PCR primer for the amplification of pSN2 to insert tandem ORFs in pSNM3-3d
repA_Rv	CTACTCCCGCATCCTCTTCAACCACTTGGA	Inverse PCR primer for the amplification of pSN2 to insert tandem ORFs in pSNM3-3d
hps_Fw_reppTT8_gib	AGGATGCGGGAGTAGGGGCCCTACCTACCG	Forward primer for amplification of the tandem ORFs in pSNM3-3d
hps_Rv_reppTT8_gib	GTGCCGTCCTGGACGCTAGGCCCCCGAGGC	Reverse primer for amplification of the tandem ORFs in pSNM3-3d

Table S4 Oligonucleotide primers used for the construction of deletion derivatives of pIOK9

Primer	Sequence (5' to 3')	Note
repSNM3-3d_1809F	TCTGGAGGGGATGGTGGTGGAAGTGGT	Forward primer to delete ORF-1 from pIOK9
repSNM3-3d_1457R	GGGTAGAGCCTACCGGGGAGGCTC	Reverse primer to delete ORF-1 from pIOK9
repSNM3-3d_2095F	GGGCTTTCCAGCCGATGCGTGTGT	Forward primer to delete ORF-2 from pIOK9
repSNM3-3d_1818R	CCCCTCCAGACTACTCCAGGGGTGGG	Reverse primer to delete ORF-2 from pIOK9

Table S5 Oligonucleotide primers used for qPCR

Primer	Sequence (5′ to 3′)	Target
Dp2039F	TCTCCCAGGAGCTTGCCATCCCCTAC	Forward primer for *polI* gene
Dp2177R	AAGAGGGTTTCCACGTAGCCCCGCTTC	Reverse primer for *polI* gene
HgR304F	GACCTGCCTGAAACCGAACTGCC	Forward primer for Hg^R gene (*hph5*)
HgR503R	TCGTCCATCACAGTTTGCCAGTCATACA	Reverse primer for Hg^R gene (*hph5*)

Table S6 Oligonucleotide primers used for the construction of xylan-utilizing vectors

Primer	Sequence (5’ to 3’)	Note
xylanase_up2_F2	GGAAACAGCTATGACCTACGGGTAGTGTTCCCCTC	Forward primer for region B (upper pathway)
half_2000_1R	GCGAACGGCCGAAATAAGCCTGCCAAAAAAGGCTACTAC	Reverse primer for region B (upper pathway)
colopy_xylan_4F	ATTTCGGCCGTTCGCGAGGTGGATC	Forward primer for region B (upper pathway)
half_2000_2R	CCCCCGCAATGCTCCTTACCTTAGACCATCTGCCAACCGC	Reverse primer for region B (upper pathway)
half_2000_3F	GGAGCATTGCGGGGGAAGGGAAAG	Forward primer for region B (upper pathway)
pSN1_Fw	GTCATAGCTGTTTCCTGTGTGAAATTGTTA	Inverse PCR primer for pIOK3_B1 and pIOK3_B2_Emp
IOK3_B_kai_F	TCAAACGAAGCCCCCTCTTAGTGC	Inverse PCR primer for pIOK3_B1
IOK3_B_kai_R	GGGGGCTTCGTTTGAGGGGTCCAGGGTAAGGGGGCG	Reverse primer for region B (upper pathway)
IOK_nega_R	GGAAACAGCTATGACGGGGTCCAGGGTAAGGGGGCG	Inverse PCR primer for pIOK3_B2_Emp
xylanase_pSN_F	AGCTGCATTAATGAACTACGGGTAGTGTTCCCCTC	Forward primer for region C (lower pathway)
xylB_pSN_R	CCGCGCGTTGGCCGATTAGGACCTCCGATAACGCTCCAA	Reverse primer for region C (lower pathway)
pHSG298Hg_1R	TTCATTAATGCAGCTGGCACGACAGGTTTC	Inverse PCR primer for pSN2K_C1
pHSG298Hg_1F	TCGGCCAACGCGCGGGGAGAG	Inverse PCR primer for pSN2K_C1
pSN_C_nqo_F	GCGGTAGTCCCCAGGGCTAGCCTCCAGGGGCCTTCTTTC	Forward primer for the nqo3 promoter for pSN2K_C2
pSN_C_nqo_R	CCACCCATTCCGCATCCCCTCCTTTCGTGCACGAAAGAATTCTTC	Reverse primer for the nqo3 promoter for pSN2K_C2
pSN_C_inverse_F	ATGCGGAATGGGTGGCTTGGTGTTTTG	Inverse PCR primer for pSN2K_C2
pSN_C_inverse_R	CCTGGGGACTACCGCTTGGTCCCG	Inverse PCR primer for pSN2K_C2
nqo_nega_F	GCACGAAAGGAGGGGTCGGCCAACGCGCGGGGAGAG	Inverse PCR primer for pSN2K_C2_Emp
nqo_reverse	CCCCTCCTTTCGTGCACGAAAGAATTCTTCTTCACGAAACC	Inverse PCR primer for pSN2K_C2_Emp

Table S7 List of vectors constructed in this study

Vectors	Notes	Accession numbers
pHSG298	*E. coli* plasmid (ColE1, Km^R), commercially available from Takara Bio	LC895503
pHSG298Hg	*E. coli* plasmid (pHSG298 derivative) carrying thermostable Hg^R gene (*hph5*)	LC895502
pIOK1	*E. coli* - *T. thermophilus* shuttle vector carrying thermostable Hg^R gene (*hph5*) and replication origin of pTthSNM1-1d	LC895501
pIOK3	*E. coli* - *T. thermophilus* shuttle vector carrying thermostable Hg^R gene (*hph5*) and replication origin of pTthSNM1-1e	LC895500
pIOK3_B2	pIOK3 carrying the region B (see **Fig. 5A**) of the xylan degradation pathway in pTthSNM4-1b	LC895498
pIOK3_Emp	A derivative of pIOK3_B2 lacking region B in pIOK3_B2	LC895497
pIOK5	*E. coli* - *T. thermophilus* shuttle vector carrying thermostable Hg^R gene (*hph5*) and replication region of pTthSNM1-7e	LC895496
pIOK7	*E. coli* - *T. thermophilus* shuttle vector carrying thermostable Hg^R gene (*hph5*) and replication region of pTthSNM1-7f	LC895495
pIOK9	*E. coli* - *T. thermophilus* shuttle vector carrying thermostable Hg^R gene (*hph5*) and replication region of pTthSNM3-3d	LC895494
pIOK9D1	pIOK9 lacking the first ORF of PEM	LC895493
pIOK9D2	pIOK9 lacking the second ORF of PEM	LC895492
pMAO1	*E. coli* - *T. thermophilus* shuttle vector carrying thermostable Km^R gene (*htk*) and replication region of pTthSNM1-1d	LC895491
pMAO3	*E. coli* - *T. thermophilus* shuttle vector carrying thermostable Km^R gene (*htk*) and replication region of pTthSNM1-1e	LC895490

pMAO5	*E. coli - T. thermophilus* shuttle vector carrying thermostable Km^R gene (*htk*) and replication region of pTthSNM1-7e	LC895489
pMAO7	*E. coli - T. thermophilus* shuttle vector carrying thermostable Km^R gene (*htk*) and replication region of pTthSNM1-7f	LC895488
pMAO9	*E. coli - T. thermophilus* shuttle vector carrying thermostable Km^R gene (*htk*) and replication region of pTthSNM3-3d	LC895487
pSN2	*E. coli - T. thermophilus* shuttle vector carrying thermostable Hg^R gene (*hph5*) and replication region of pTT8	LC895486
pSN2B	pSN2B equipped with PEM of pIOK9	LC895485
pSN2K_C1	pSN2K carrying the region C (see **Fig. 5A**) of the xylan degradation pathway in pTthSNM4-1b	LC895484
pSN2K_C2	A derivative of pSN2K_C1 carrying the nqo3 promoter	LC895483
pSN2K_C2_Emp	A derivative of pSN2K_C1 lacking region C in pSN2K_C2	LC895482

```
TthSNM11_25190 (pIOK1)    1 METPIRPGSFHNEEARKTLASILNFLQKDRALGERALHLWAAMHGLDPEEALALARSG------EVPPSPA-----PEAPP-PPSGSAVQEGTFALQHSVSTPLREDDPGLPLEVAGGRC    108
TthSNM17_24640 (pIOK7)    1 METPIRPGSFHNEEARKTLASILNFLQKDRALGERALRLWAAMHGLDPEEALALAQSGEAPTSQEAPPSPA-----PEAPYTPPSGSAVQEGTFALQHSVSTPLREDDPGLPLEVAGGRC    115
TthSNM11_25290 (pIOK3)    1 M-VPERFSEAHRNPENQALQISLEALRK-----------------LGPAAERLARRLG------LVVDPPAEGAAFPEVPSTPPSGSTLQNGTFALQHSVPTPLRGDDSGPFLEV-GGRC     95
TthSNM17_24530 (pIOK5)    1 M-VPERFSEAHRNPENQALQISLEALRK-----------------LGPAAERLARRLG------LVVDPPAEGAAFPEAPP-PPSGSAVQEGTFALQHSVSTPLREDDPGLPLEVAGGRC     95
TthSNM33_25510 (pIOK9)    1 M-VPERFSEAHRNPENQALQISLEALRK-----------------LGPAAERLARRLG------LVVDPPAEGAAFPEVPSTPPSGSTLQNGTFALQHSVSTPLREDDPGLPLEVAGGRC     96
                            * .*.*....*.......*...*..*.*                 *.*.........*      .....**.....**.* .*****..*.*********.****.**.*..***.****

TthSNM11_25190 (pIOK1)  109 PPSGFTPGRWDNECCTATPAPMIAVQDGENRALAWVRRLLQQTLAETPEDDPRHQALQDLLALAEEKGLEAGPLRLLLLLAALGRRYLRERFGPKAARKAGQAVFHLPVDLVASFLGVHR    228
TthSNM17_24640 (pIOK7)  116 PPSGFTPGRWDNECCTATPKPMMTVQDGENRALAWVRRLLQQTLAETPEDDPRRQALQDLLALAEEKGLEAGPLRLLLLLAALGRRYLRERFGPKAARKAGQAVFHLPVDLVASFLGVHR    235
TthSNM11_25290 (pIOK3)   96 SLSGIEPGRWESECCTATPKPMMTVQDGENRALAWVRRLLQQTLAETPEDDPRRQALQDLLALAEEKGLEAGPLRLLLLLAALGRRYLRERFGPKAARKAGQAVFHLPVDLVASFLGVHR    215
TthSNM17_24530 (pIOK5)   96 PPSGFTPGRWDNECCTATPKPMMTVQDGENRALAWVRRLLQQTLAETPEDDPRRQALQDLLALAEEKGLEAGPLRLLLLLAALGRRYLRERFGPKAARKAGQAVFHLPVDLVASFLGVHR    215
TthSNM33_25510 (pIOK9)   97 PPSGFTPGRWDNECCTATPAPMIAVQDGENRALAWVRRLLQQTLAETPEDDPRHQALQDLLALAEEKGLQAGPLRLLLLLAALGRRYLRERFGPKAARKAGQAVFHLPVDLVASFLGVHR    216
                            ..**..****..*******.**..****************************.****************.**************************************************

TthSNM11_25190 (pIOK1)  229 TTVWRWAGPLEEAGLLRSKTHVATAVRGGEAQNLNTGTVWAVRLKPGRARFEHGDLTHPWRDLAKDLEEGRTAFRVIYPKGKRRKKGDQTERLRPERVSLELLVKWTLGIREVPALDFLR    348
TthSNM17_24640 (pIOK7)  236 TTVWRWAGPLEEAGLLRSKTHVATAVRGGEAQNLNTGTVWAVRLKPGRARFEHGDLSHPWRDMAKDLEEGRTAFRVIYPKGKRRRKGDQTERLRPERVSLELLVKWTLGIREVPALDFLR    355
TthSNM11_25290 (pIOK3)  216 TTVWRWAGPLEEAGLLRSKTHVATAVRGGEAQNLNTGTVWAVRLKPGRARFEHGDLTRPWRDMAKDLEEGRTAFRVIYPKGKRRRKGDQTERLRPERVSLELLVKWTLGIREVPALDFLR    335
TthSNM17_24530 (pIOK5)  216 TTVWRWAGPLEEAGLLRSKTHVATAVRGGEAQNLNTGTVWAVRLKPGRARFEHGDLSHPWRDMAKDLEEGRTAFRVIYPKGKRRRKGDQTERLRPERVSLELLVKWTLGIREVPALDFLR    335
TthSNM33_25510 (pIOK9)  217 TTVWRWAGPLEEAGLLRSKTHVATAVRGGEAQNLNTGTVWAVRLKPGRARFEHGDLTHPWRDMAKDLEEGRTAFRVIYPKGKRRKKGDQTERLRPERVSLELLVKWTLGIREVPALDFLR    336
                            ********************************************************..****.********************.***********************************

TthSNM11_25190 (pIOK1)  349 PAPTENPTIEAAFLLAEMEAQDRPTVIDLLSERMAHELGDPHSRRFYAGLLWKVVEGKLSPHALVHAYHRARAAVREGYARRGGAFLQHLLK----AAA                         443
TthSNM17_24640 (pIOK7)  356 PAPTENPTIEAAFLLAEMEAQDRPTVIDRLAERMAHELNDPHSRRFYAGLLWKVVEGKLSPHALVHAYHRARAAVREGYARRGGAFLQHLLKDLLKAAA                         454
TthSNM11_25290 (pIOK3)  336 PAPTENPTIEAAFLLAEMEAQDRPTVIDLLSERMAHELGDPHSRRFYAGLLWKVVEGKLSPHALVHAYHRARAAVREGYARRGGAFLQHLLE----AAA                         430
TthSNM17_24530 (pIOK5)  336 PAPTENPTIEAAFLLAEMEAQDRPTVIDRLAERMAHELNDPHSRRFYAGLLWKVVEGKLSPHALVHAYHRARAAVREGYARRGGAFLQHLLKDLLKAAA                         434
TthSNM33_25510 (pIOK9)  337 PAPTENPTIEAAFLLAEMEAQDRPTVIDLLSERMAHELGDPHSRRFYAGLLWKVVEGKLSPHALVHAYHRARAAVREGYARRGGAFLQHLLK----AAA                         431
                            ****************************.*.*******.****************************************************.    ***
```

Figure S1. Amino acid sequence alignment of the REP proteins used to construct the shuttle vector. Residues conserved in all sequences are indicated by an asterisk (*), and frequently occurring residues (residues present in three or four of the five sequences) are indicated by dots.

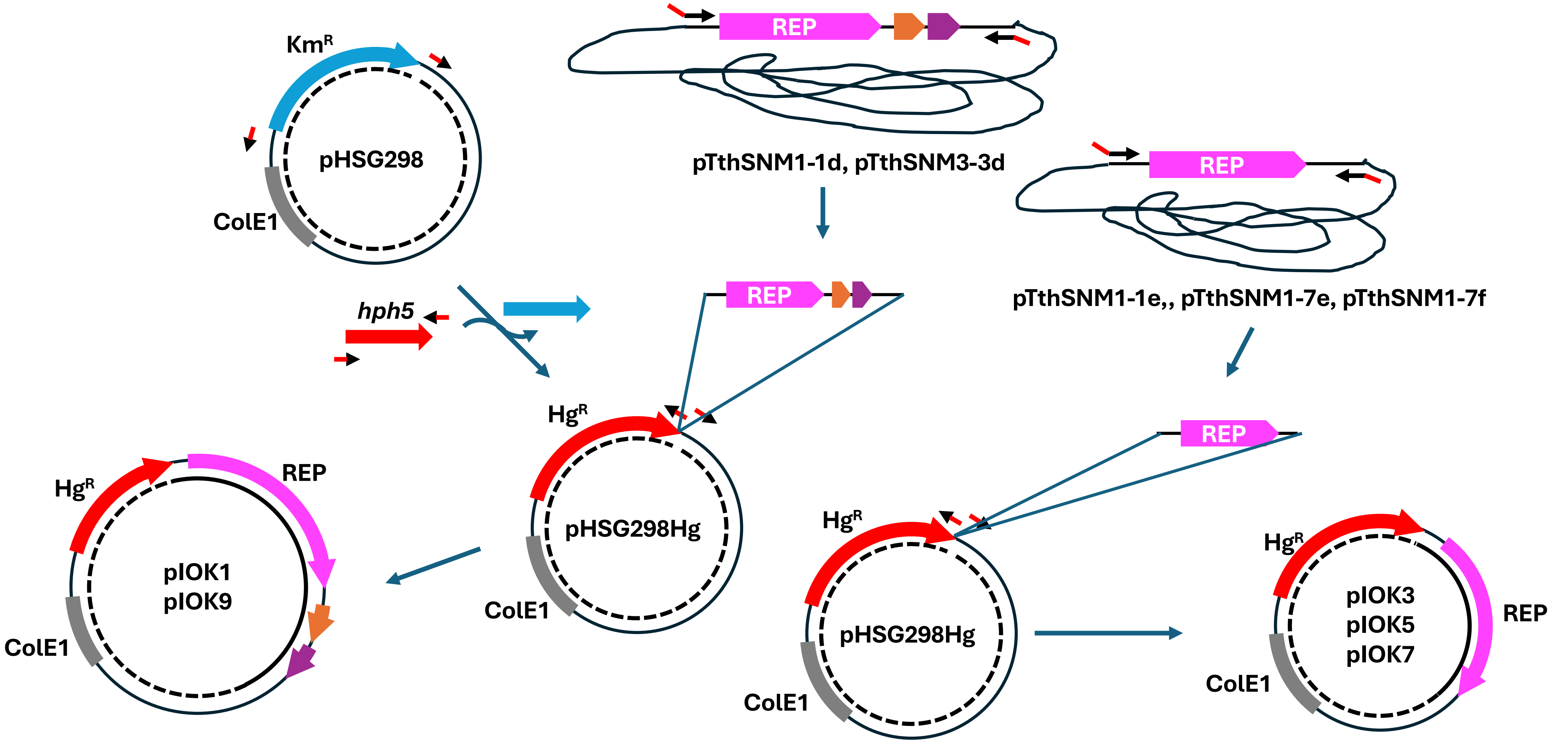

Figure S2. Plasmid construction workflow. A commercially available *E. coli* plasmid vector pHSG298 was used as a backbone plasmid throughout. To use the vector in *T. thermophilus*, a selectable marker and replicon were cloned into the vector. First, the kanamycin resistance gene (Km^R) in pHSG298 was replaced by a thermostable hygromycin resistance gene *hph5.* Next, a putative REP protein coding gene and its flanking regions were PCR-amplified from *T. thermophilus* plasmids and cloned downstream of the *hph5* gene mediated by Gibson assembly. Arrows in black are the regions that hybridize to the target sequence, while those in red are the overlap regions for Gibson assembly. Solid lines in the inner circle represent the part derived from pHSG298 and *hph5*, while the hatched lines are the part derived from *T. thermophilus* plasmids.

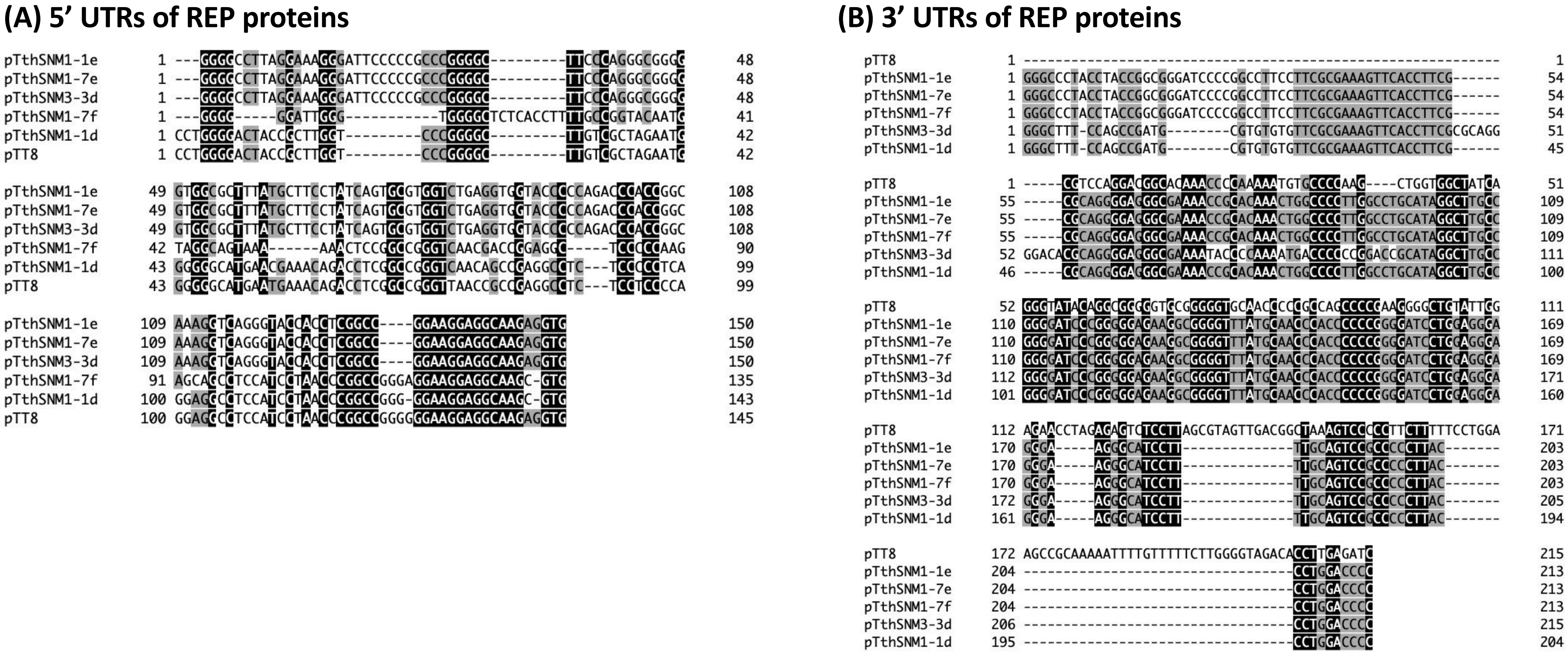

Figure S3. Nucleotide sequence alignment of the 5' and 3' untranslated regions of the REP protein used to construct the shuttle vector. **(A)** The 5' untranslated regions of the REP protein. The GTG sequence at the 3' end is the start codon for the REP protein. **(B)** The 3' untranslated regions of the REP protein. Residues conserved across all sequences are shown in white letters on a black background, and frequently occurring residues (present in four or five of the six sequences) are shaded.

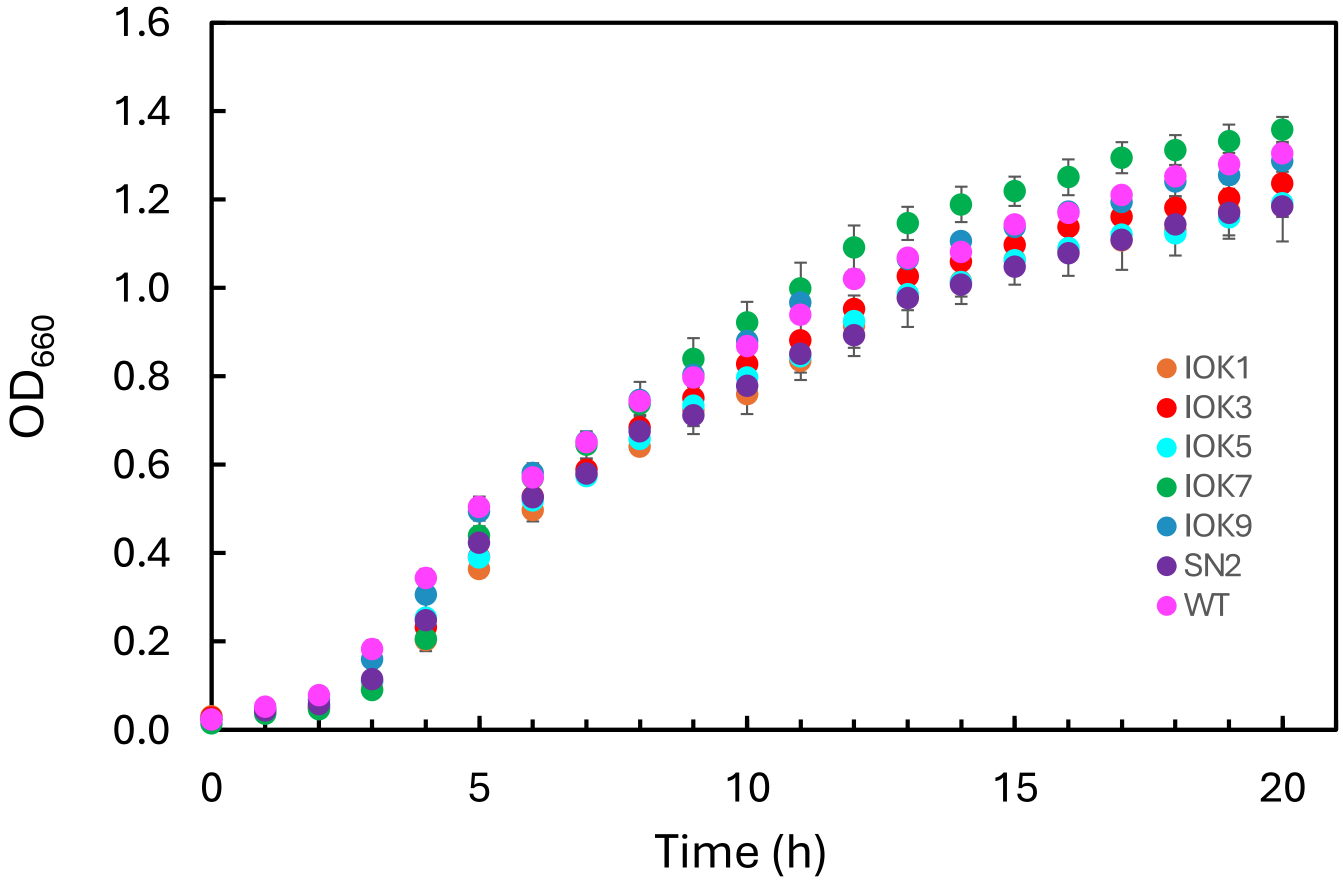

Figure S4. Growth curves of *T. thermophilus* HB27 and its mutant transformed with pIOK vectors.

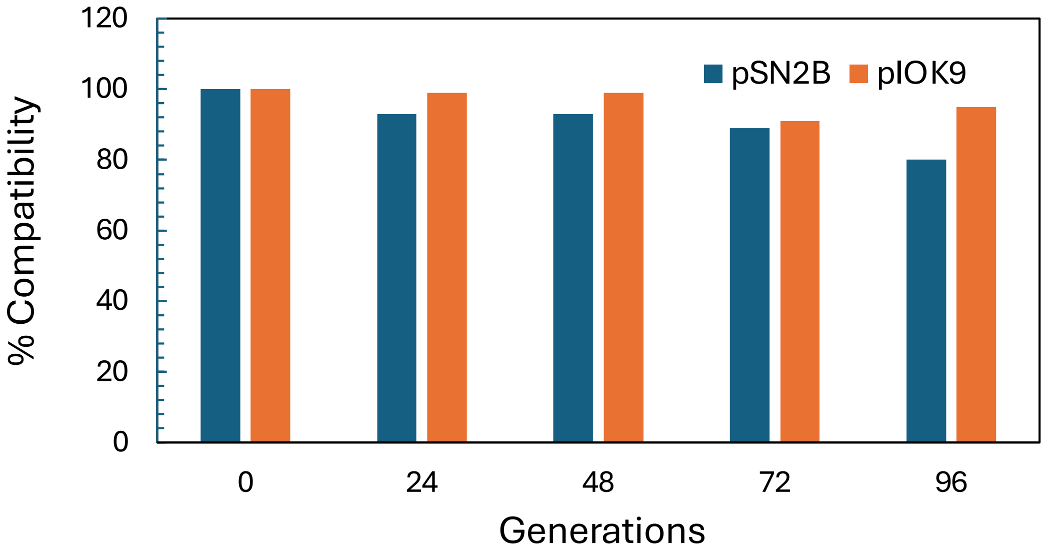

Figure S5. Persistence of pSN2B and pIOK9 in the presence of pMAO1 (Km^R). Shuttle vectors pMAO1 (Km^R) and pSN2B (Hg^R) or pMAO1 (Km^R) and pIOK9 (Hg^R) were co-introduced into *T. thermophilus* HB27, and double transformants were selected on TT/Km/Hg plates. For each combination, a single colony was inoculated in 5 mL of TT/Km medium and cultivated at 65°C. Every 24 h, a portion of the culture was inoculated into a fresh 5 mL TT/Km medium at a 1:1000 dilution, while another portion was appropriately diluted (~$1/10^5$) and spread on TT/Km plates. After overnight cultivation at 65 °C, a total of 100 colonies were randomly selected from the plates and replica-plated onto TT/Km and TT/Km/Hg plates. The number of colonies on TT/Km was taken as 100% and the number of colonies on TT/Km/Hg was used to calculate the compatibility rate (represented as % compatibility in the Y-axis). The doubling time for growth was approximately 1 h, and thus the number of generations was considered equal to the cultivation time (h).
